# Vimentin activation in early apoptotic cancer cells errands survival pathways during DNA damage inducer CPT treatment in colon carcinoma model

**DOI:** 10.1038/s41419-019-1690-2

**Published:** 2019-06-13

**Authors:** Souneek Chakraborty, Aviral Kumar, Mir Mohd Faheem, Archana Katoch, Anmol Kumar, Vijay Lakshmi Jamwal, Debasis Nayak, Aparna Golani, Reyaz Ur Rasool, Syed Mudabir Ahmad, Jedy Jose, Rakesh Kumar, Sumit G Gandhi, Lekha Dinesh Kumar, Anindya Goswami

**Affiliations:** 1 Academy of Scientific & Innovative Research (AcSIR), CSIR-Indian Institute of Integrative Medicine, Jammu, 180001 India; 2 Cancer Pharmacology Division, CSIR-Indian Institute of Integrative Medicine, Jammu, 180001 India; 3 Cancer Biology, CSIR-Centre for Cellular & Molecular Biology, Hyderabad, 500007 India; 4 Plant Biotechnology Division, CSIR-Indian Institute of Integrative Medicine, Jammu, 180001 India; 5 School of Biotechnology, Shri Mata Vaishno Devi University, Katra, 182320 India

**Keywords:** Metastasis, Chemotherapy

## Abstract

Epithelial to mesenchymal transitions (EMT) is a preparatory process for cancer cells to attain motility and further metastasis to distant sites. Majority of DNA damaging drugs have shown to develop EMT as one of the major mechanisms to attain drug resistance. Here we sought to understand the resistance/survival instincts of cancer cells during initial phase of drug treatment. We provide a tangible evidence of stimulation of EMT factors in *Apc* knockout colorectal carcinoma model. Our results implied that CPT-treated *Apc* knockout cohorts depicted increased pro-invasive and pro-survival factors (Vimentin/p^ser38^Vimentin & NFκB). Moreover, by cell sorting experiment, we have observed the expression of Vimentin in early apoptotic cells (AnnexinV positive) from 36 to 48 h of CPT treatment. We also observed the expression of chimeric Sec-AnnexinV-mvenus protein in migrated cells on transwell membrane recapitulating signatures of early apoptosis. Notably, induction of Vimentin-mediated signaling (by CPT) delayed apoptosis progression in cells conferring survival responses by modulating the promoter activity of NFκB. Furthermore, our results unveiled a novel link between Vimentin and ATM signaling, orchestrated via binding interaction between Vimentin and ATM kinase. Finally, we observed a significant alteration of crypt-villus morphology upon combination of DIM (EMT inhibitor) with CPT nullified the background EMT signals thus improving the efficacy of the DNA damaging agent. Thus, our findings revealed a resistance strategy of cancer cells within a very initial period of drug treatment by activating EMT program, which hinders the cancer cells to achieve later phases of apoptosis thus increasing the chances of early migration.

## Introduction

Epithelial to mesenchymal transition (EMT) is one of the earliest events in cancer cell metastasis. EMT can be viewed as a preparatory mechanism where the cells acquire the mesenchymal phenotype, by undergoing cytoskeletal rearrangements, to gain motility for further metastasis^1^. Intermediate filaments (IF) play a vital role during EMT progression by maintaining cellular stiffness^2^. Out of the six major IFs, Vimentin (type 3 IFs) is considered as the most important facilitator for mesenchymal cellular stiffness. Vimentin gets expressed in epithelial cells only when EMT is activated otherwise they solitary express keratin as a major IF. Apart from its cytoskeletal role, phosphorylated counterpart of Vimentin acts as signaling agent during EMT and interacts with numerous proteins to execute strong cellular survival responses^3^.

Although EMT is reported to contribute towards drug resistance^4,5^, the mechanism by which EMT facilitates cancer cells to acquire resistance lacks adequate research. Whether EMT is a collateral event during drug resistance or a programmed strategy for the cancer cells to escape the drug-induced stresses, is an emerging field of study. Majority of drug development programs in cancer aim at selective elimination of cancer cells by modulating apoptotic mechanisms like DNA damage, oxidative stress, etc. Families of drugs which damage DNA and hinder its repair mechanisms are widely used in cancer therapeutics, with majority of FDA-approved drugs belonging to this category^6–8^. But from the very beginning, these class of drugs confront the problem of resistance^4,9–13^. For that reason, there is an urgent need to investigate the cause of failure of this class of DNA damaging drugs.

In this research, we provide strong evidences of activation of EMT and survival factors during initial phases of treatment with DNA damaging agent Camptothecin in *Apc* floxed colorectal carcinoma model as well as in colorectal/lung carcinoma cell lines. Notably, we found the expression of Vimentin that coexists along with early apoptotic population further hinders apoptotic progression unless otherwise accelerated in presence of EMT inhibitor (Di-indoyl Methane). Additionally, we have described the role of ATM kinase in Vimentin phosphorylation thus modulating its pro-survival and pro-migratory function.

## Results

### CPT treatment confers Vimentin activation and EMT induction in colon carcinoma

**Fig. 1:**
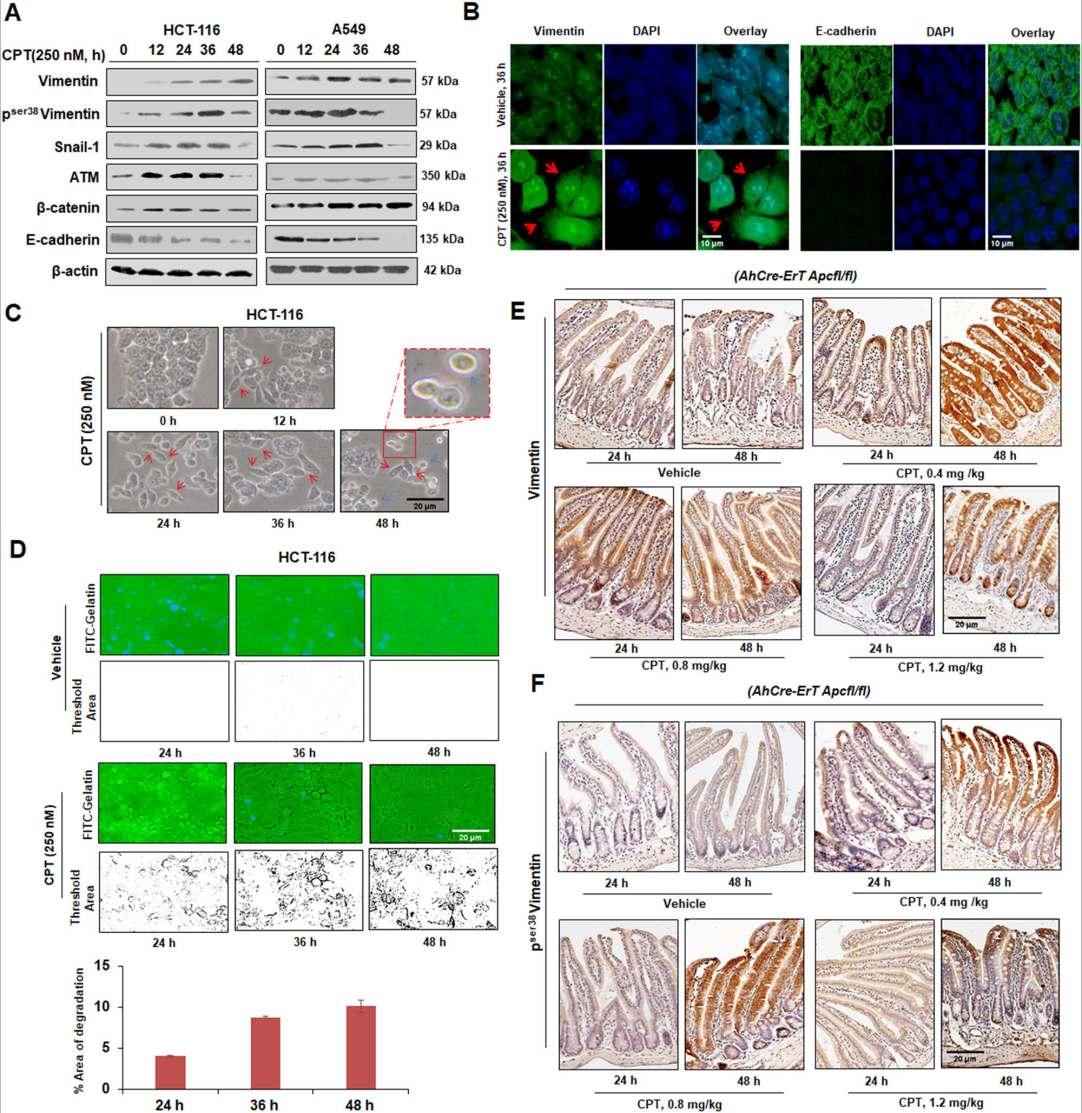
Activation of EMT and apoptosis in Camptothecin-mediated DNA damage response. **a** HCT-116 and A549 cells were treated with 250 nM of CPT for 0, 12, 24, 36, and 48 h and checked for the expression of Vimentin, p^ser38^Vimentin, Snail-1, ATM, β-catenin, and E-cadherin through western blot analysis. β-actin was used as loading control. **b** Immunocytochemistry was performed in HCT-116 cells treated with vehicle and CPT (250 nM) for 36 h for checking the expression of Vimentin, E-cadherin (green fluorescence). Nuclear staining was done with DAPI containing mounting media. Magnification of the images = ×63, **c** Analysis of the morphological features of HCT-116 cells through microscopic observation after exposure of cells to CPT for increasing time points (magnification = ×20). **d** Cells were treated with CPT (250 nM) for 24, 36, and 48 h along with vehicle and tested for their ability to degrade gelatin matrix and invadopodia formation through FITC-gelatin degradation assay. Blue stains indicate nuclear staining through DAPI mounting media. Images were taken at ×20 magnification. Bar graph showing the threshold area of degradation quantified through Image j analysis (*n* = 3, error bars ± s.d.). **e**, **f** Induced *AhCre-ErT* *Apcfl/fl* mice were treated with CPT (0.4, 0.8, and 12.2 mg/kg) for 24 and 48 h and the dissected intestinal tissues were sectioned and subjected to immunohistochemistry, to analyze the expression of Vimentin and p^ser38^Vimentin. Images were taken at ×20 magnification

The typical role of EMT in drug resistance and stemness acquisition in cancer cells is recently understood but little is known about how cancer cells survive by activating EMT with an aim to circumvent apoptosis/anoikis^5^. In our preliminary studies, distinct morphological changes of epithelial cells were observed when treated with DNA damaging agents (data not shown). Rationally, we sought to examine the effects of CPT-mediated DNA damages on EMT activation in cancer cells. Western blot analysis showed a gradual increase in the expression of EMT specific marker-Vimentin, in three different epithelial cell lines (HCT-116, Sw-620, and A549) when treated with increasing concentrations of CPT (ranging from 50 to 250 nM) for 36 h (Supplementary Fig. 1A). Similar results were obtained following treatment with increasing concentrations of 5-Flurouracil (5-FU), Doxorubicin, and Cis-platin (Supplementary Fig. 1B and 2). Since 250 nM CPT is optimal for apoptotic induction^14^ and given the fact that Vimentin was adequately expressed at this concentration, 250 nM CPT was employed for further time-dependent studies. When HCT-116 and A549 cells were subjected to 250 nM CPT (0–48 h), diminishing E-cadherin expression followed by gradual up-regulation of Vimentin levels achieved indicating the induction of EMT in these cells (Fig. 1a). Although, Vimentin phosphorylation is an important event for both EMT persistence and cell survival^3^, CPT-treated cells exhibited a steep increase in p^ser38^Vimentin expression up to 36 h, then dropped sharply afterwards (Fig. 1a). While a steady amplification of Snail-1, ATM, and β-catenin expression were obtained by CPT treatment (12–36 h), however, ATM and Snail-1 expression diminished at 48 h (Fig. 1a). The immunocytochemistry results validated our immunoblots experiments. The localization of Vimentin in the nucleus, cytosol, and in migrating structures (indicated by red arrow heads) additionally confirmed its role in survival responses^15^. Conversely, the gradual disappearance of E-cadherin from the cellular surface was also noticed (Fig. 1b). The bright field microscopy revealed that the epithelial morphology of vehicle-treated HCT-116 cells disappeared within 24 h of CPT treatment and majority of cells attained mesenchymal morphology at 36 & 48 h (Fig. 1c). Interestingly, the apoptotic population was predominantly noted at 48 h (blue arrow head for apoptotic bodies and red arrow head for mesenchymal cells). To reason the cellular protrusion like structures in Fig. 1b (red arrows), we evaluated the invasive capability of the CPT-treated cells by FITC-gelatin degradation assay revealing a significant increase in gelatin degradation following CPT treatment, implying the acquisition of invasiveness by HCT-116 / A549 cells (Fig. 1d, Supplementary Fig. 1C).

*Apc* conditional knock out murine colorectal carcinoma model was used to evaluate EMT initiation by CPT-induced DNA damage in vivo. This model mimics early tumor development along the entire length of the small intestine^16,17^. Mice cohorts were treated with three different concentrations of CPT (0.4, 0.8, and 1.2 mg/kg) for 24 and 48 h immediately after induction. An early appearance of “crypt progenitor” phenotype in the CPT-treated mice was found (0.4 mg/kg) compared to vehicle. However, at higher concentration of CPT (1.2 mg/kg), the villus and crypt boundaries started to restore (Supplementary Fig. 3A). The immunohistochemistry analyses of intestinal sections obtained from CPT-treated mice (0.4 mg/kg) revealed a significant rise in Vimentin and p^ser38^Vimentin expression compared to control cohorts manifesting EMT induction in vivo (Fig. 1e, Supplementary Fig. 3B). At higher CPT concentrations (0.8 and 1.2 mg/kg) Vimentin expression was more as compared to vehicle but gradually diminished as compared to the 0.4 mg/kg CPT-treated mice. (Fig. 1e, f). But the expression of p^ser38^Vimentin was elevated in both 0.4 & 0.8 mg/kg concentrations at 48 h, which eventually decelerated following 1.2 mg/kg of CPT treatment (Fig. 1f, Supplementary Fig. 3C). Notably, the curing dose of CPT (1.2 mg/kg) was unable to completely nullify the associated EMT which might hamper apoptosis. These results clearly suggest that the tendency of DNA damage induced by chemotherapeutic agents accelerates EMT and pro-metastatic signaling which might facilitate the escape of the cancer cells during early stages of chemotherapeutic regime resulting in chemo-resistant secondary tumors^18^.

### Simultaneous occurrence of EMT and apoptosis in CPT-treated cells

**Fig. 2:**
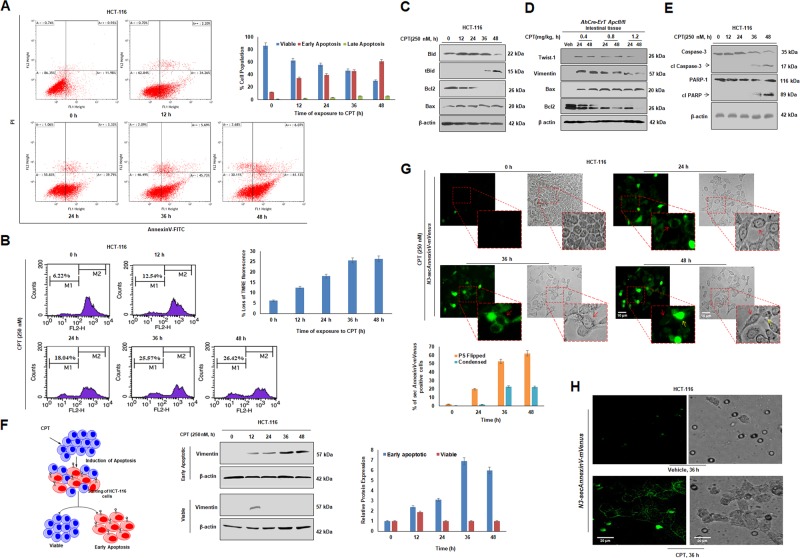
Co-existence of EMT and early-apoptosis in same population of Camptothecin treated cells. **a** Cells were treated with CPT for 0, 12, 24, 36, and 48 h, tagged with AnnexinV-FITC, propidium iodide and analyzed through flow cytometry for onset of apoptosis. Bar graph showing quantification of cells in various phases of apoptosis (*n* = 3, error bars ± s.d.). **b** HCT-116 cells treated with CPT (250 nM) for 0, 12, 24, 36, and 48 h, and subjected to FACS analysis for identification of disruption of mitochondrial membrane potential by TMRE staining. Bar graph depicting loss of TMRE fluorescence indicating loss of mitochondrial membrane potential. **c** Western blot analysis was performed to study the expression pattern of Bid, Bcl_2_, Bax by exposing HCT-116 cells to 250 nM CPT for 0, 12, 24, 36, and 48 h. β-actin was used as loading control. **d** Western blot analysis of *Apc* floxed intestinal tissue treated with CPT (0.4, 0.8, and 1.2 mg/kg) for 24 and 48 h was performed to analyze the expression of Twist-1, Vimentin, Bax, and Bcl_2_. β-actin was used as loading control. **e** HCT-116 treated with 250 nM CPT for 0, 12, 24, 36, and 48 h was subjected to immunoblot analysis to check the expression of Caspase-3 and PARP-1. **f** HCT-116 cells were treated with CPT (250 nM) for 0, 12, 24, 36, and 48 h, stained with AnnexinV-FITC and PI and sorted using MoFlo cell sorter for the early apoptotic and viable cells (schematic). The sorted cells were then analyzed for the expression of Vimentin by immunoblot analysis; β-actin was used as loading control. Bar graph represents the densiometric analysis of expression of Vimentin in early apoptotic and viable population of sorted cells; (*n* = 3, error bars ± s.d.). **g** HCT-116 cells were transfected with *N3-secAnnexinV-mVenus* construct and then treated with 250 nM of CPT for 24, 36, and 48 h. Images were taken at ×20 magnification. Red arrows indicate cells with fluorescence bordering the cells and yellow arrows indicate cells having sufficient fluorescence throughout the cells. Bar graph depicting number of PS (Phosphatidyl serine) flipped cells *vs* condensed cells showing bright fluorescence. Cell counting was performed in Image j software (*n* = 3, error bars ± s.d). **h** HCT-116 cells transfected with *N3-secAnnexinV-mVenus* construct were subjected to transwell migration assay and treatment was given with 250 nM of CPT for 36 h. The migrating cells were processed further and observed under fluorescence microscope for detecting the fluorescence from chimeric AnnexinV-mVenus protein

Apoptosis involves multistep processes and each step encompasses unique cytological and molecular signatures. While flipping of phosphatidyl serine (PS) to outer membrane leaflets is one of the early events in apoptosis^19–21^, damage of chromatin reticulum or DNA fragmentation occurs at later stages^22^.To examine whether any apoptotic parameters prevailed in the CPT-treated cells, HCT-116 and A549 cells were treated with CPT (0–48 h) followed by AnnexinV-PI staining. FACS analysis data clearly revealed that with the increase in the time of CPT treatment there was a gradual decrease in the percentage of viable cells with simultaneous increase in the percentage of early apoptotic cells. Interestingly, early apoptotic population was almost equivalent to the viable population at 36 h of CPT treatment (~47% = viable = early apoptotic population) in HCT-116 cells. Similarly, CPT-treated A549 cells showed equivalent percentages of viable and early apoptotic fractions at 36 h, indicating that EMT induction goes hand in hand with apoptotic stimulation upon CPT treatment (Fig. 2a, Supplementary Fig. 4).

Since initial disruption of mitochondrial transmembrane potential (ΔΨm) occurs quite early in apoptotic cascades^19^, we sought to evaluate whether there is any loss of ΔΨm upon CPT exposure in a time-dependent manner. TMRE fluorescence quantification by FACS depicted a gradual increase in the low fluorescent TMRE fraction of cells (M1 quadrant), indicating periodical loss of ΔΨm upon CPT treatment (Fig. 2b). Bcl_2_ family proteins play a vital role in cytochrome c release by disrupting the transmembrane of mitochondria. The western blotting analysis unearthed significant augmentation of pro-apoptotic markers -Bax and truncated Bid, with downmodulation of pro-survival protein –Bcl_2_ in presence of CPT (Fig. 2c). The in vivo data also revealed an inverse relationship between Bax and Bcl_2_ expression in agreement with our in vitro results (Fig. 2d). As early stages of apoptosis are quite flexible and a lot of survival proteins can manipulate these stages^20,23^, we investigated more committed stages of apoptosis in time-dependent CPT treatment. Since, prominent cleavage of Caspase-3 and PARP-1 was observed at 48 h of CPT treatment (Fig. 2e), this time point (48 h) could be considered as a junction where the cells fully commit to the apoptotic fate. To extensively investigate the relation between EMT and apoptosis, AnnexinV-FITC and propidium iodide (PI) stained HCT-116 cells were sorted into early apoptotic and viable fractions using MoFlo cell sorter. The immunoblot analysis of the early apoptotic fraction revealed an increased Vimentin expression 12 h onwards, whereas the viable cells expressed Vimentin only at 12 h of CPT treatment (Fig. 2f). The presence of Vimentin in early apoptotic cells validated co-existence of apoptosis and EMT in cells threatened by a DNA damaging agent. We sought to understand the same phenomena by transfecting CPT-treated HCT-116 cells with *N3-secAnnexinV-mVenus* construct^24^ developed specifically to tag apoptotic cells with flipped PS. Interestingly, cells expressing AnnexinV-mVenus (green fluorescence bordering the cells) depicted mesenchymal morphology in the bright field images (red arrows; Fig. 2g), underscoring co-existence of mesenchymal and apoptotic cells. The bright fluorescent cells (yellow arrows) were observed majorly at 48 h seemed to be a conglomeration of apoptotic bodies (Fig. 2g)^21^. Further, non-transfected CPT-treated HCT-116 cells (12–36 h) were supplemented with the conditional media from *N3-secAnnexinV-mVenus* transfected cells. Accordingly, the conditional media containing secretory AnnexinV-mVenus chimeric protein interacted with the flipped Phosphatidylserine of non-transfected cells contemplating the green fluorescence validating our hypothesis (Supplementary Fig. 5). Interestingly, the transfected *N3-secAnnexinV-mVenus* cells traversed the Boyden chamber membrane confirming those apoptotic cells (due to CPT treatment) possessed enhanced migratory behavior (Fig. 2h) whereas vehicle (DMSO) treated chambers were devoid of any migratory cells (Fig. 2h). The cell scattering results also supported similar observations (Supplementary Fig. 6). Collectively, these above results demonstrate that during initial phases of drug treatment both early apoptotic as well as EMT phenotypes co-exist together within the same population of cells.

### EMT activation triggered pro-proliferative responses during CPT treatment in vitro and in vivo

**Fig. 3:**
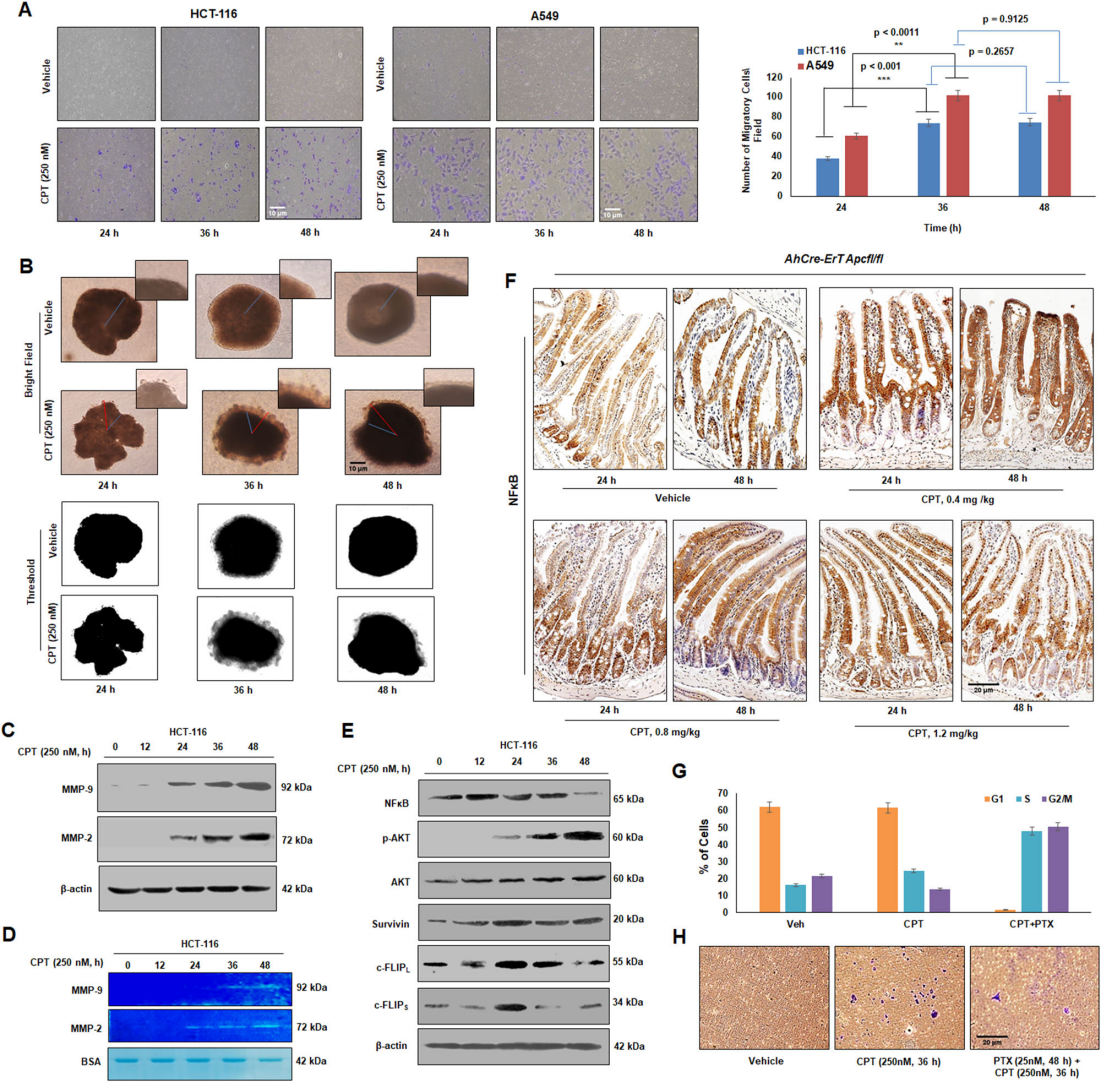
Camptothecin-mediated activation of pro-proliferative responses. **a** HCT-116 and A549 cells were treated with CPT (250 nM) for 24, 36, and 48 h along with their respective vehicle controls; cells were analyzed for their invasion capability through Boyden chamber assay system. Images were taken under an inverted microscope (Nikon Eclipse 200) at ×10 magnification. Bar graphs represent the average number of migrated cells per field (*n* = 3, error bars ± s.d.); ***p < 0.001, **p < 0.0011, *p* = 0.9125 and *p* = 0.2657. **b** Spheroids were prepared with HCT-116 cells embedded in ECM matrix by hanging drop method and treated with indicated concentration of CPT for given time points. Migration of cells from the core of the spheroids was observed (upper panel) under inverted microscope at ×20 magnification. The corresponding threshold images (lower panel) were prepared by using Image j software. **c** HCT-116 cells treated for indicated time pulse with 250 nM of CPT; whole cell lysates were analyzed for the expression of MMP-2 and MMP-9 protein through immunoblot analysis. **d** Conditional media was collected from the above experiment and subjected to gelatin zymography to examine the gelatinase activity of MMP-2 and MMP-9; bovine serum albumin (BSA) was taken as a loading control. **e** HCT-116 cells were treated with indicated concentration of CPT for 0, 12, 24, 36, and 48 h; the whole cell lysates were subjected to western blotting for the analysis of NFκB, p-AKT, AKT, Survivin, and c-FLIP_S/L_ proteins. **f** CPT (0.4, 0.8, and 1.2 mg/kg) treatment was orally given to induced *AhCre-ErT Apcfl/fl* mice for 24 and 48 h and the harvested intestinal tissues were subjected to immunohistochemistry analysis of NFκB protein. **g** HCT-116 cells were treated with vehicle, CPT (250 nM) and Paclitaxel (25 nM). Paclitaxel treatment was administrated 12 h before the treatment of CPT. Cycle analysis was performed through flow cytometry and the bar graph represents percentage of cells in different phases of cell cycle, (*n* = 3, error bars ± s.d.). **h** Boyden chamber assay was performed to check the invasion ability of HCT-116 cells treated with above set of treatment conditions

Oncogenic EMT not only acts as a preparatory mechanism for the cells to gain motility for further metastasis but also allows them to evade cell death by initiating survival signaling^25,26^. To study the effects of DNA damage on cell migration, Boyden chamber assay was performed where CPT-treated HCT-116 and A549 cells robustly traversed the membrane (Fig. 3a). The 3D spheroids assay with HCT-116 cells showed distinct dispersion of cells from the spheroid periphery into the surrounding Matrigel upon treatment with CPT (24 h/36 h/48 h) as compared to control (Fig. 3b). Moreover, the cells displayed the maximum migration at 36 h as evident by the highest concentric radius (Red line- migratory front; Blue line- inner spheroid core). Further, a steady augmentation of MMP-2 and MMP-9 expression in HCT-116 cells was noticed following CPT treatment accompanied with increased gelatin degradation (by MMP-2 and MMP-9) in time-dependent manner (Fig. 3c, d).

Next, we sought to evaluate whether there were any concurrent perturbations in cellular pro-survival signals due to CPT treatment. The western blot results clearly depicted an escalated cFLIP_L/S_ expression up to 24 h following a sharp attenuation at 48 h in presence of CPT, while Survivin showed a biphasic expression pattern (Fig. 3e). Given that NFκB is a master regulator of cell survival responses^27,28^, its expression amplified at 12 h and the level was maintained up to 36 h followed by a steep down-modulation at 48 h (Fig. 3e). The immunohistochemistry analysis of CPT-treated *Apc* floxed mice unveiled high NFκB expression in 0.4 mg/kg CPT-treated cohorts compared to their respective vehicle controls at 24 and 48 h (Fig. 3f). The NFκB expression decreased significantly in 1.2 mg/kg CPT-treated cohorts compared to 0.4 & 0.8 mg/kg (Fig. 3f, Supplementary Fig. 7).

Both EMT and apoptosis has been linked with cell cycle phases and cells in G_1_ arrest showed preference towards migration and survivability compared to G_2_M arrested cells where apoptosis is favored^29^. In the current situation, when cells were blocked in G_2_M phase by paclitaxel (25 nM) along with CPT, a significant inhibition in cell migration was observed along with diminished Vimentin expression (Fig. 3g, h and Supplementary Fig. 8). All these results collectively demonstrate a profound activation of survival responses due to the stimulation of EMT cascades.

### Induction of Vimentin confers apoptotic resistance and promotes survival responses due to CPT treatment

**Fig. 4:**
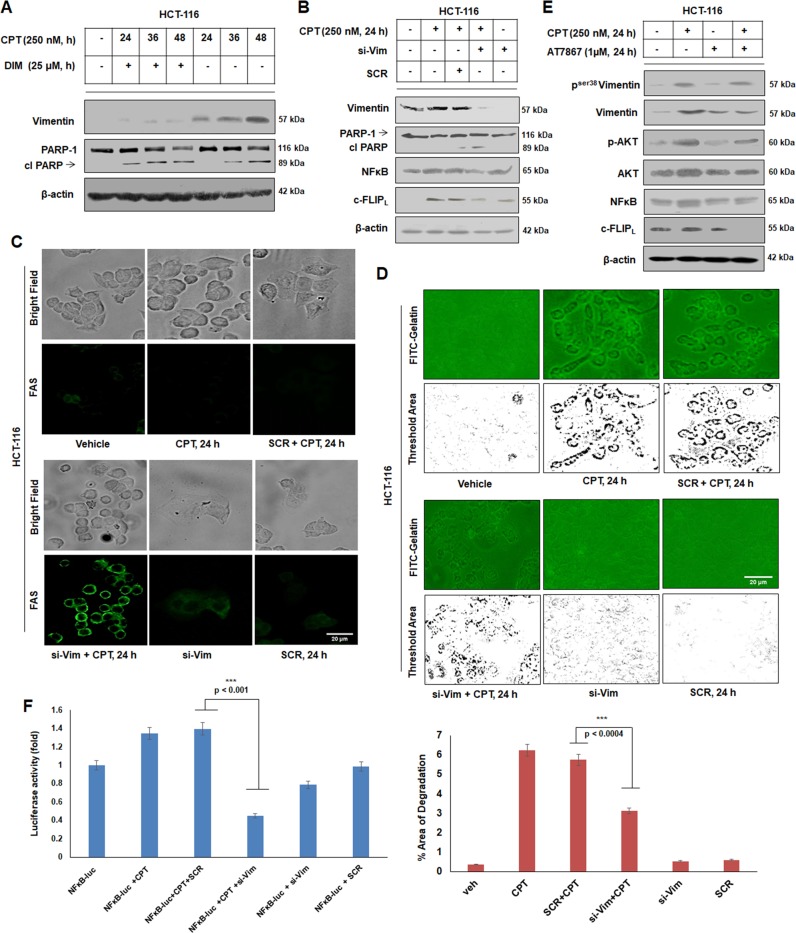
Induction of Vimentin hinders the progression through apoptosis. **a** HCT-116 cells were either treated with vehicle, CPT (250 nM) and CPT (250 nM) + DIM (25 µM) for indicated time points; whole cell lysates were prepared and subjected to western blot analysis of Vimentin and PARP-1 protein. **b** Cells were either transfected with vehicle, CPT (250 nM), SCR + CPT (250 nM), si-Vimentin plus CPT (250 nM) and si-Vimentin for 24 h; whole cell lysates were employed for western blot analysis of Vimentin, PARP-1, NFκB, and cFLIP. **c** Immunocytochemistry was performed for the indicated treatment conditions to examine the expression and trafficking of FAS ligand in HCT-116 cells. Images were taken by using Floid Cell Imaging Station; magnification ×20. **d** Similar set of treatment conditions were used to check the cells capability for gelatin matrix degradation through FITC-gelatin degradation assay. Imaging was performed using Floid Cell Imaging Station at ×20 magnification. The images were further analyzed and quantified for threshold area of degradation by Image j software and represented in bar graphs (*n* = 3, error bars ± s.d.); ***p < 0.0004. **e** HCT-116 cells were either treated with vehicle, CPT (250 nM), AT7867 (1 µM) and combination of both for 24 h; whole cell lysates were subjected to western blot analysis for the expression of p^ser38^-Vimentin, Vimentin, p-AKT, AKT, NFκB, and cFLIP_L_. **f** HCT-116 cells were either transfected with vector, NFκB-luc alone and/or treated with NFκB-luc + CPT (250 nM), NFκB-luc + CPT (250 nM) + SCR, NFκB-luc + si-Vimentin + CPT (250 nM), NFκB-luc + si-Vimentin, and NFκB-luc + SCR in 96 well plates for 24 h; luciferase activity was measured using Dual-Glo Luciferase Assay system (Promega). Normalization was done with luciferase activity of vector (*n* = 3, error bars ± SD). ***p < 0.001

In order to understand the function of Vimentin in modulating apoptosis, Di-indolyl methane (DIM)- a potent EMT blocker^30–32^ was used to nullify the CPT-induced EMT. Interestingly, EMT-blockade by DIM shifted the PARP-1 cleavage pattern to 24 and 36 h compared to CPT (without DIM) treatment where prominent PARP-1 cleavage was obtained only at 48 h (Fig. 4a). This shift in the PARP-1 cleavage pattern could be attributed to the decelerated Vimentin levels in presence of DIM thereby promoting cell death. The significance of Vimentin induction as an anti-apoptotic factor was further confirmed in CPT-treated HCT-116 cells, wherein, transient knock-down of Vimentin resulted in enhanced PARP-1 cleavage at 24 h in comparison to scramble transfected cells (Fig. 4b). Further, we observed the attenuation of survival proteins *viz*. cFLIP and NFκB in CPT-treated cells compared to scrambled controls wherein both cFLIP and NFκB expressions elevated gradually. Since, cFLIP possesses specific role in blocking FAS trafficking, which is one of the major mediators of apoptosis, the cells were subjected to Vimentin si-RNA knockdown followed by ICC analysis. Vimentin knockdown resulted in loss of cFLIP expression leading to significant FAS trafficking to the membrane (Fig. 4c). Additionally, Vimentin knockdown alleviated the invasive capability of CPT-treated HCT-116 cells as observed in the FITC-gelatin degradation assay (Fig. 4d and Bar graph).To examine the consequences of AKT inhibition, a major upstream activator of Vimentin^33^, the cells were treated with AT-7867 (allosteric AKT inhibitor) leading to a significant down-modulation of p^Ser38^Vimentin, cFLIP and NFκB in CPT-treated lane along with AT7867 compared to lane treated with CPT only indicating a direct correlation between survival factors and Vimentin (Fig. 4e). Since, NFκB expression was downmodulated in CPT plus si-Vimentin condition, we hypothesized that Vimentin could have a role in controlling NFκB transcription at its promoter level. We transfected the HCT-116 cells with NFκB luciferase reporter construct and analyzed NFκB transcriptional activity through a Dual-Glo Luciferase Assay System. The luciferase activity of each sample was normalized by the luciferase activity of the PGL2 vector. The CPT-treated cells demonstrated a significant increase in luciferase activity that attenuated significantly in si-Vimentin condition (around threefold) (Fig. 4f). Together, these results suggest that Vimentin induction has an integrative role in controlling residual survival responses and progression through apoptosis by directly or indirectly regulating the transcriptional activation of NFκB protein.

### ATM kinase activation is essential to maintain Vimentin related endeavors during DNA damage condition

**Fig. 5:**
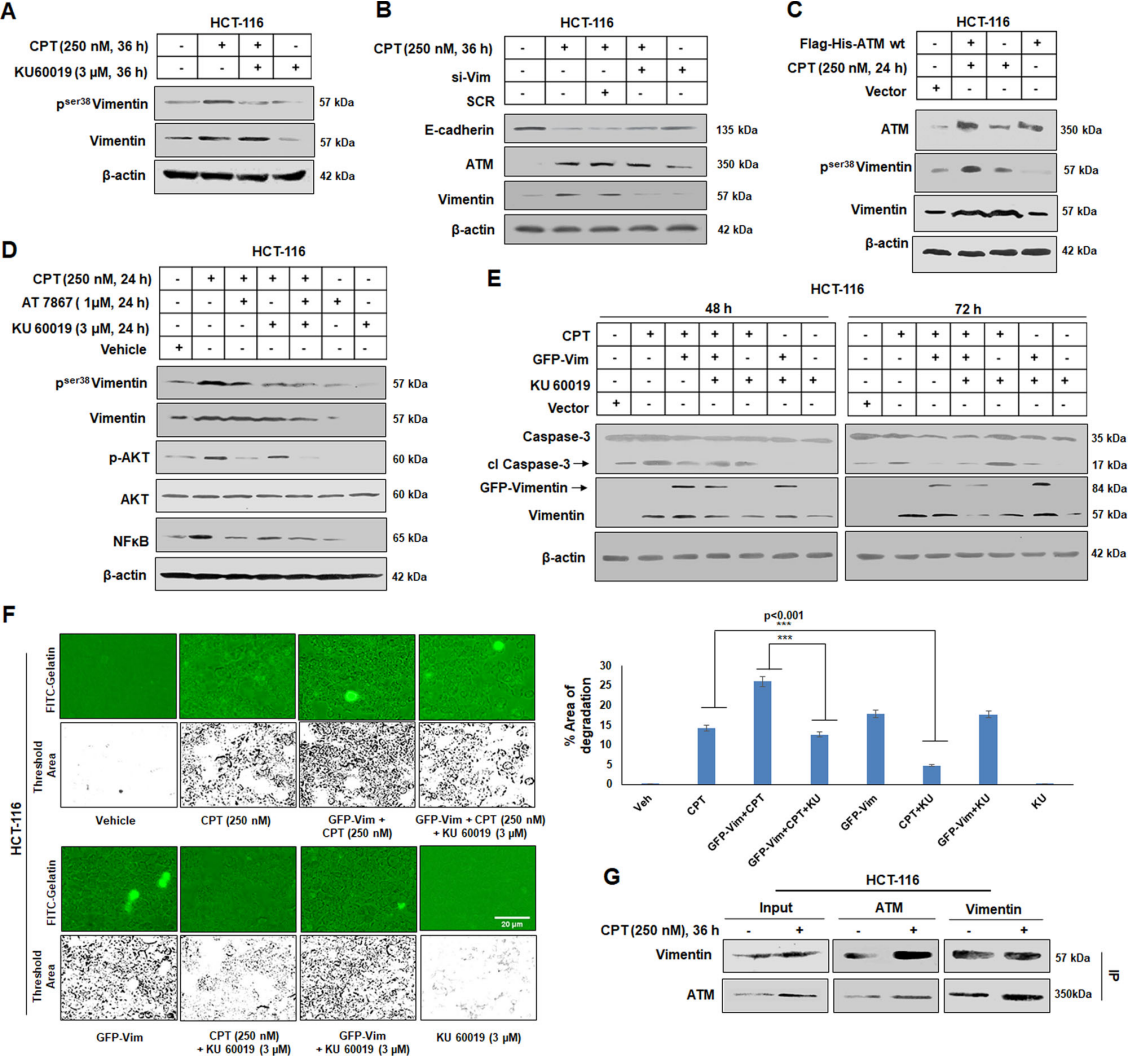
ATM-mediated control of Vimentin and its downstream responses. **a** HCT-116 cells were treated with vehicle, CPT (250 nM), KU60019 (3 µM), KU60019 (3 µM) + 250 nM CPT for 36 h; whole cell lysates were prepared and western blotting was performed to determine the expression of p^ser38^ Vimentin and total Vimentin. **b** Cells were either treated with Vehicle, CPT, SCR + CPT (250 nM), si-Vimentin + CPT (250 nM), and si-Vimentin for 36 h; western blotting was performed to analyze the expression of Vimentin, ATM, and E-cadherin. **c** HCT-116 cells treated with Vector, Flag-His-ATM wt + CPT (250 nM), CPT (250 nM) and Flag-His-ATM wt for 24 h and subjected to western blot analysis to evaluate the expression of ATM, p^ser38^Vimentin, and total Vimentin. β-actin was used as loading control. **d** Western blot analysis was performed after HCT cells were treated with vehicle, CPT (250 nM), CPT (250 nM) + AT 7867 (1 μM), CPT (250 nM) + KU60019 (3 μM), CPT (250 nM) + AT 7867 (1 μM) + KU60019 (3 μM), AT 7867 (1 μM), and KU60019 (3 μM). The expression of p^ser38^Vimentin, total Vimentin, p-AKT, AKT, NFκB were analyzed keeping β-actin as loading control. **e** HCT-116 cells were treated with Vector, CPT (250 nM), CPT (250 nM) + GFP-Vimentin, CPT (250 nM) + GFP-Vimentin + KU60019 (3 µM), CPT (250 nM) + KU60019 (3 µM), GFP-Vimentin + KU60019 (3 µM), and KU60019 (3 µM) for 48 and 72 h and subjected to western blot analysis to understand the expression of Caspase-3 and Vimentin, β-actin was used as loading control. **f** HCT-116 cells (upper panel) were seeded onto gelatin-FITC coated glass coverslips and treated with Vehicle, CPT (250 nM), CPT (250 nM) + GFP-Vimentin, CPT (250 nM) + GFP-Vimentin + KU60019 (3 µM); lower panel: GFP-Vimentin, CPT (250 nM) + KU60019 (3 µM), GFP-Vimentin + KU60019 (3 µM), and KU60019 (3 µM) for 36 h and the slides were observed in Floid imaging station at ×20 magnification. Bar graph describes the percentage threshold area of degradation analyzed by Image j software. (*n* *=* 3, error bars ± SD); ***p < 0.001. **g** Immunoprecipitation analysis of ATM with Vimentin after HCT-116 cells were treated with Vehicle and CPT (250 nM) for 36 h

Recent evidences suggest that several components of DNA damage and repair pathway activate EMT inducing factors^34,35^. ATM kinase, a DNA damage sensor, reportedly phosphorylates and activates Snail-1, a major transcription factor controlling EMT associated genes^34^. We explored how ATM kinase plays a central role in orchestrating Vimentin during CPT treatment in HCT-116 cells. We have already demonstrated that the expression of ATM kinase was well synchronized with the p^ser38^Vimentin as well as Snail-1 levels in both HCT-116 and A549 cells (Fig. 1a). Hence, we hypothesized that ATM kinase might play an active role in phosphorylating Vimentin, in DNA damage condition. Therefore, when HCT-116 cells were treated with KU60019, a potent ATM kinase inhibitor, the expression of p^ser38^Vimentin declined significantly in presence of CPT (Fig. 5a). However, following silencing of Vimentin by siRNA, we could not find any significant change in expression of ATM kinase compared to corresponding control (Fig. 5b). Further, the role of ATM kinase in phosphorylating Vimentin was analyzed by transiently overexpressing ATM kinase in HCT-116 cells with pcDNA3.1(+) Flag-His-ATM wt. We observed significant rise in the level of p^ser38^Vimentin expression in lane overexpressing ATM kinase treated with 250 nM CPT as compared to lane treated with CPT only (Fig. 5c). Seeing that AKT plays a vital role in phosphorylating Vimentin^33^, in Fig. 4e, we observed that the expression of p^ser38^Vimentin was still in considerable quantity in condition where AKT was blocked by AT-7867. This indicates that other kinases also play a role in phosphorylating Vimentin. Although, in Fig. 5a, c, we found that ATM kinase aids in phosphorylating Vimentin at Serine 38 position, we now sought to understand the importance of ATM in Vimentin phosphorylation over AKT in CPT-mediated DNA damage condition. We, therefore, employed both AKT and ATM inhibitors (AT-7867 & KU60019) together as well as separately in CPT-treated conditions. Interestingly, we observed that the expression of p^ser38^Vimentin diminished sharply in KU60019 + CPT lane as compared to AT-7867 + CPT. Moreover, the expression of p^ser38^Vimentin reduced more profoundly where both the kinases were blocked in presence of 250 nM CPT (Fig. 5d). Additionally, the expression of NFκB also decreased upon blocking ATM/AKT separately as well as in the lane where both the kinases were blocked simultaneously as compared to CPT alone condition. Next, we sought to understand the integrative role of ATM in promoting the anti-apoptotic function of Vimentin. Intriguingly, by inhibiting ATM, cleaved caspase-3 augmented sharply in ectopic Vimentin lane, which otherwise depleted in GFP-Vimentin lane in presence of CPT (Fig. 5e). Subsequently, the implication of ATM in Vimentin-mediated migration of HCT-116 cells treated with CPT was examined and the results showed a significant rise in cell migration in Vimentin over-expressed condition. Further, ATM inhibition by KU60019 drastically affected the migration of cells (Fig. 5f, bar graph). Since, Vimentin in its phosphorylated form has been reported to interact with various kinases and based on our previous results (Fig. 5a, c), we speculated that Vimentin and ATM kinase could directly/indirectly interact with each other. To examine our hypothesis, co-immunoprecipitation assay clearly unveiled the interaction between ATM and Vimentin in CPT-treated/untreated samples (Fig. 5g). Cumulatively, these results clearly implied that ATM-mediated Vimentin activation during DNA damage responses is vital to exaggerate Vimentin function through halting of apoptotic progression and stimulate cellular migration.

### Inhibition of CPT-induced EMT by DIM confers anti-metastatic potential of DIM in vivo

**Fig. 6:**
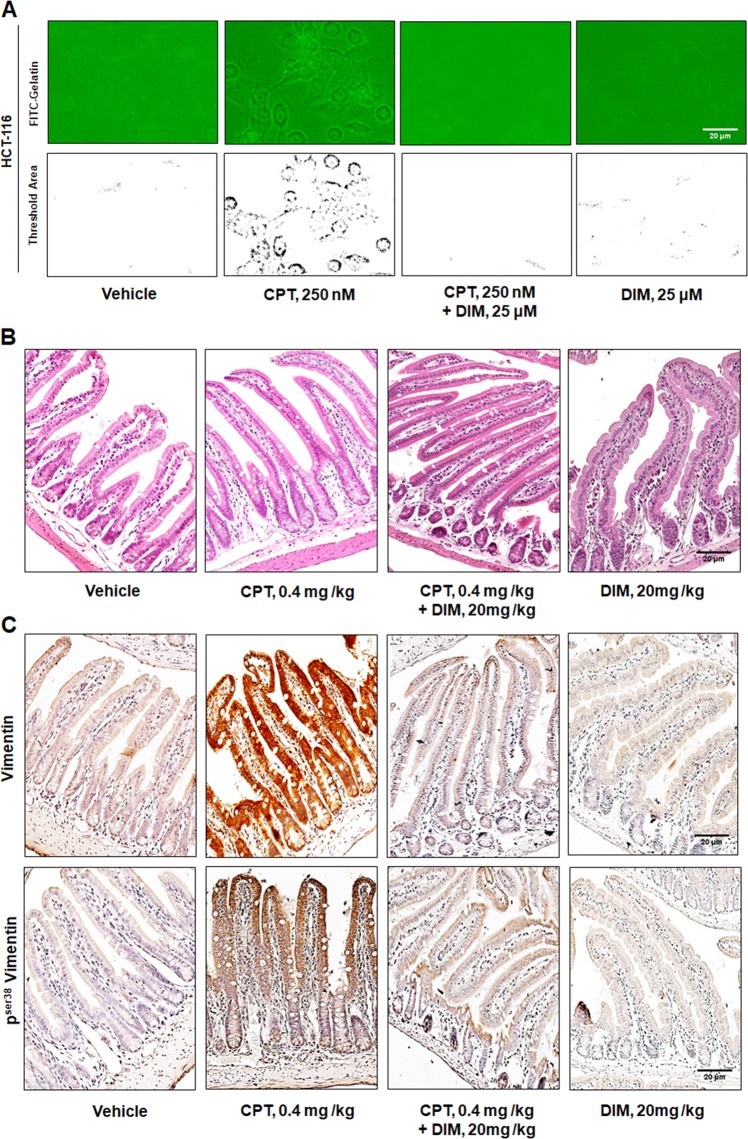
DIM mediated abrogation of CPT-induced carcinogenesis and EMT. **a** HCT-116 cells seeded onto FITC-gelatin coated coverslips were treated with Vehicle, 250 nM CPT, 250 nM CPT + 25 μM DIM, and 25 μM DIM for 36 h and the slides were observed in fluorescence microscope at ×20 magnification. **b** Hematoxylin and Eosin staining of intestinal tissue obtained from induced *AhCre-ErT Apcfl/fl* treated with Vehicle, 0.4 mg/kg CPT, 0.4 mg/kg + 20 mg/kg DIM, and 20 mg/kg DIM for 48 h. Images were taken at ×20 magnification. **c** Immunohistochemistry analysis of Vimentin and p^ser38^ Vimentin was performed on intestinal tissues obtained from *AhCre-ErT Apcfl/fl* mice treated with Vehicle, 0.4 mg/kg CPT, 0.4 mg/kg + 20 mg/kg DIM, and 20 mg/kg DIM for 48 h. Images were acquired in ×20 magnification

Next, the study with DIM in previous section encouraged us to further understand the potential of this drug, i.e. to increase the therapeutic efficacy of DNA damaging agent by abrogating the co-parallel EMT activation. In gelatin degradation assay, a significant abrogation of cellular invasion was noticed when HCT-116 cells were co-treated with CPT and DIM (25 μM) compared to alone CPT-treated condition (Fig. 6a). Di indoyl methane is a potential antimetastatic drug reported to exhibit high bioavailability and low toxicity^36^; these properties of DIM led us to conduct a combinatorial study with CPT in *Apc* knock out murine model. The results clearly indicated a drastic alteration of crypt progenitor phenotype in CPT (0.4 mg/kg) in combination with DIM (20 mg/kg) as compared to CPT alone with 48 h (Fig. 6b). In this combination approach, both the expression of Vimentin and p^ser38^Vimentin was significantly diminished but was otherwise substantially amplified in CPT-treated cohorts (Fig. 6c and Supplementary Fig. 9). Together, our results suggest a combinatorial therapeutic approach of Di indoyl methane with CPT enhanced the efficacy and potency of CPT by nullifying the associated EMT phenotypes.

## Discussion

Majority of chemotherapeutic drugs induces DNA damage responses (DDR) signaling cascade leading to cell death. However, dysregulation of DDR pathways might result in hypersensitivity or resistance of tumors to therapy. Identification of the molecular mechanisms underlying this phenomenon is essential for enhancing the potential of cancer therapy^37^. The activation and stabilization of Snail-1 by ATM kinase and its further involvement in promoting metastasis poses vital questions concerning the simultaneous progression of apoptosis and EMT in cancer cells^34,38^. Many other groups also reported links between DDR and premature activation of pro metastatic and pro survival responses^39,40^ but evidences are not concrete. Incitement of EMT, indeed poses cellular survivability, but the activation of apoptotic machinery by CPT can’t be overlooked^14^. In the present study we provide evidences that CPT treatment from 0–36 h activates signatures of EMT and cellular survival along with early apoptotic factors in cancer population both in vitro (HCT-116, SW620, A549 cells) and in vivo (Apc floxed colorectal carcinoma model) conditions. But as the time progressed to 48 h the survival factors got attenuated and Caspase-3/PARP-1 cleavage became more profound.

**Fig. 7:**
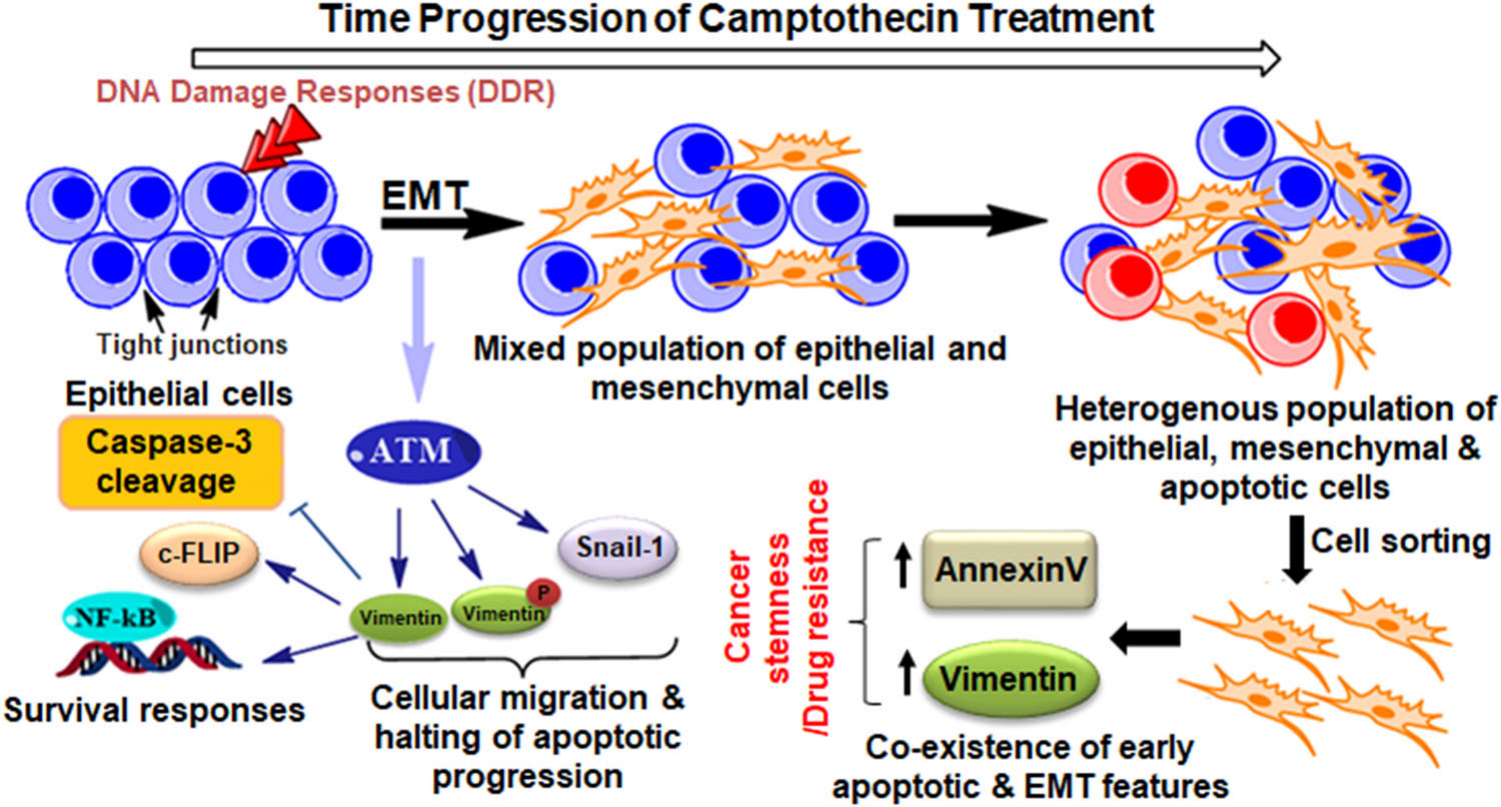
**Schematic representation of the proposed mechanism of action of the study depicting the co-existence of early-apoptotic and EMT features with time progression of Camptothecin treatment**

Both EMT and apoptosis were thought to be mutually exclusive fates involving distant machinery in a cell, until recently Ham et al. and David et al. clearly drew a connection between these two processes^41,42^. To shed light on this emerging concept of unification of these two evolutionarily conserved processes we found the existence of mesenchymal marker (Vimentin) in early apoptotic fraction of HCT-116 cells and inversely we have shown the binding of AnnexinV-mVenus to mesenchymal cells exhibiting migratory potential. Expression of Vimentin solely in 12 h time point within the viable population is undoubtedly a unique feature and currently our laboratory is envisaging on this interesting finding. Next step was to understand the mutual correlation of these two evolutionary significant biological processes-apoptosis and EMT, for that we employ Di-indole methane, a potential anti-metastatic molecule to nullify the associated EMT and we also silenced Vimentin by using si-RNA. In both the cases we have shown a shift in committed stages of apoptosis towards earlier time points and mitigation of pro-survival and pro-metastatic responses. The results also describe a positive signaling link between Vimentin and NFκB, which was feebly reported earlier by Huang et al^43^. further fortifying the less understood survival role of Vimentin apart from its well-studied structural functions (Fig. 7).

ATM kinase is a major DNA damage sensor and upon activation it halts the cell cycle and stimulate effectors of DNA repair pathways^44^. Additionally, activation of ATM kinase triggers several cellular survival responses and EMT which can be detrimental for the patients undergoing chemotherapy leading to further drug resistance^45,46^. Therefore, this research elucidates a novel signaling link between ATM kinase and Vimentin as we have shown that both these proteins physically interacts with each other and activated ATM kinase aids in maintain Vimentin in its phosphorylated form in ser38 region. Moreover, the results demonstrate that ATM plays a pivotal role in facilitating Vimentin to execute its functions related to hindering apoptosis as well as boosting invasion.

Finally, the anomalous activation of EMT and cellular survival pathways during initial phases of drug treatment challenges the basic structure of chemotherapy by DNA damaging agents and there is a grave need of designing new therapeutic approaches to prevent these collateral processes so that the tumor cells achieve committed stages of apoptosis efficiently resulting in successful therapeutics eliminating the problem of drug resistance.

## Materials and methods

### Cell culture and reagents

HCT-116 (colon carcinoma) and A549 (lung carcinoma) were purchased from American Type Culture Collection (ATCC, Manassas, VA, USA). HCT-116 and A549 cells were maintained in RPMI-1640 medium supplemented with 10% fetal bovine serum (Gibco, Carlsbad, CA, USA). HEPES, penicillin, streptomycin, sodium pyruvate and sodium bicarbonate were purchased from Sigma-Aldrich, St Louis, Missouri, US. The cells were maintained in a humified CO_2_ incubator (New Brunswick, Galaxy 170R) at 37 °C with 5% CO_2_. CPT and 5FU were purchased from Merck, Kenilworth, NJ, U.S.A. and inhibitors z-VAD-FMK and KU60019 were obtained from Santa Cruz Biotechnology Inc (Santa Cruz, CA) and Merck (NJ, U.S.A) respectively. Di indoyl Methane (DIM) was purchased from TCI chemicals (Tokyo, Japan).

### Immunoblot analysis

Immunoblot analysis was performed according to the standard procedure described previously by our group^47^. The list of antibodies and their dilutions are provided in Supplementary Table S1.

### Immunocytochemistry

Immunocytochemistry experiments were carried out according to the established protocol of our laboratory^48^. The list of antibodies and their dilutions are provided in Supplementary Table S1. Images were taken using confocal microscope—Leica TCS SP8 (Leica Microsystem, Mannheim, Germany).

### In situ fluorescent gelatin degradation assay

The matrix gelatin degradation assay was performed to assess the formation of invadopodia or podosomes. Following treatment, cells were analyzed by fluorescence microscope (Floid Cell Imaging Station) according to the method previously described^48^.

### Detection of mitochondrial membrane potential

Mitochondrial membrane potential was analyzed by FACS (BD FACS CALIBUR, New Jersey, USA). 1 × 10^6^ HCT-116 cells were seeded in 60 mm petri dishes and after overnight incubation the cells were treated with CPT for indicated time points. Upon termination the cells were trypsinized and 10^6^ cells/ml suspension was prepared by serum free media, 400 nM of TMRE dye was added and incubated for 20 min at 37 °C. After incubation cells were washed with PBS and resuspended in it, subsequently the TMRE fluorescence was measured by flow cytometer at excitation/emission = 459/575.

### AnnexinV-FITC/PI analysis

HCT-116 cells were seeded at 5 × 10^5^ cells/well in 35 mm dishes and incubated for overnight at 37 °C and 5% CO_2_. Next day, the cells were treated with vehicle and CPT (250 nM) for indicated time points. The AnnexinV-FITC experiments were performed using the AnnexinV-FITC Apoptosis Detection Kit according to the manufacturer’s manual (Sigma-Aldrich, St Louis, Missouri, US). Briefly, cell pellets were re-suspended in 600 μL binding buffer (10 mM HEPES [N-2- hydroxyethylpiperazine-N-2–ethane sulfonic acid], 140 mM NaCl, and 2.5 mM CaCl_2_, pH 7.4), and stained with 6.0 µl Annexin V-FITC and 10 µl propidium iodide staining solution for 20 min at RT in dark. The samples were successively analyzed by FACS (BD FACS Aria II) using BD Diva software.

### Cell sorting of AnnexinV-FITC positive cells

Post staining with AnnexinV-FITC (as described in the above section), sorting of HCT-116 cells was performed in MoFlo cell sorter (MoFlo-Astrios EQ, Beckham-Coulter, Indianapolis, IN, US) as per the manufacturer’s instruction. Further, the sorted cells were collected in HBSS (Hank’s Balanced Salt Solution) from the sorter; then thoroughly rinsed with PBS and subsequently suspended in RIPA buffer for total protein extraction followed by western blotting.

### Sub cloning of *sec AnnexinV-mVenus* into human pEGFP-N3 vector

Specific primers SecA5mV(Fw) and SecA5mV(Rev) **(**Integrated DNA Technology, lowa, USA), were used to obtain a 1.7 kb amplicon carrying AnnexinV-mVenus fusion protein with a secretary tag at 5^/^ end, from the template vector pTol2pA2-3.5ubb:secHsa.AnnexinV-mVenus,crya:mCherry (gifted by Tjakko van Ham, Department of clinical genetics: Erasmus MC), in a PCR reaction with total volume of 50 μl containing 5 μl buffer (10×), 3 μl MgSO_4_ (25 mM), 5 μl dNTPs (2 mM), 5 μl each primer (3 μM), 0.5 μl of *Vent* DNA polymerase (New England BioLabs, Ipswich, Massachusetts, US), under the following conditions: 5 min at 95 °C, 35 cycles of 20 s at 95 °C, 20 s at 50 °C and 2 min at 75 °C. The amplicon and pEGFP-N3 target plasmid (Human expression vector) were double digested using EcoR1 and BamH1 along with Tango 10× buffer (Fermentas-Thermo Scientific) restriction enzymes. Digested products were gel eluted using Qia-quick gel extraction kit (Qiagen, The Netherlands) following manufacturer’s protocol. The eluted product was ligated into the pre-digested pEGF-N3 plasmid using T4 DNA Ligase (Thermo Fisher Scientific, Waltham, Massachusetts, US) by incubating it at 37 °C for 4–5 h. The ligated product was further transformed in DH5α competent cells. Transformed cells were spreaded on LB plates (with 50 μg/ml kanamycin) and incubated overnight at 37 °C for further growth. The colonies were screened through colony PCR using same specific set of primers SecA5mV (Fw) and SecA5mV (Rev). Plasmids were isolated from positive colonies and were sequenced.

### Transient transfection with *N3-secAnnexinV-mVenus* construct

5 × 10^3^ HCT-116 cells were seeded per well in an 8 well chamber slide and transfections were performed in Opti-MEM media (Thermo-Fisher Scientific, Waltham, Massachusetts, United States) using lipofectamine 3000 (Life Technologies, Carlsbad, California, USA) according to the manufacturer’s instructions with some modifications. Subsequently, the cells were treated with CPT for indicated time points and observed under fluorescence microscope (Floid imaging station, Thermo Fisher Scientific, USA).

### Transient transfection with *GFP-Vimentin* construct & *pcDNA3.1(+) Flag-His-ATM wt*

*GFP-Vimentin* and *pcDNA3.1(+) Flag-His-ATM wt* constructs were kind gift from Howard Donninger (Assistant Professor, Department of Medicine, University of Louisville School of Medicine) & Tej K. Pandita (Professor of Radiation Oncology, Houston Methodist Weill Cornell Medical College). 1 × 10^6^ HCT-116 cells were seeded in 60 mm petri dishes and transfected with GFP-Vimentin and Flag-His-ATM wt constructs by using lipofectamine 3000. Transection efficiency was determined by both microscopy (for GFP-Vimentin) and western blotting.

### Transwell migration assay

The cell invasion through Matrigel was carried out with the BD Biocoat Tumor Invasion Assay System (BD Biosciences, Bedford, MA) according to the manufacturer’s instruction and as described previously^49^. To understand the co-existence of migration and apoptosis in HCT-116 cells, *N3-secAnnexinV-mVenus* transfected cells were seeded in transwell chambers (24 well format). Post CPT treatment (36 h), the cells were fixed in methanol and observed under a fluorescence microscope (×20 magnification).

### Cell scatter assay

HCT-116 cells were seeded in a 6 well plate at a seeding density of 1000–2000 cells per well and kept for 4–5 days to develop small discrete colonies. Subsequently, the cells were transfected with N3-secAnnexinV-mVenus construct. Further, the transfectants were treated with 250 nM CPT for 36 h. After termination, the cells were washed with PBS and fixed with 4% paraformaldehyde, and then observed under fluorescence microscope (×20 magnification).

### 3D spheroid migration assay

Microscopic spheroids of HCT- 116 cells were generated by hanging drop method^50^. Once visible spheroids were obtained, they were shifted to 1.5 mm Eppendorf tubes and centrifuged at 300–400 rpm for 5 min. After washing the spheroids with PBS, they were embedded into Matrigel (BD MATRIGEL, BD Biosciences). Once the embedded mixture was solidified, RPMI media was added carefully. Subsequently, CPT treatment was given for indicated time points. The migration of cells from the core of the spheroids was observed under inverted microscope-20× magnification (Nikon Eclipse 200) and threshold images were created by using Image j software (ver.1.50i, National Institute of Health, USA).

### Gelatin Zymography

Gelatin Zymography was performed in order to understand the gelatinase activity of MMP-2 following a previously standardized method^48^.

### Cell cycle analysis by flow cytometry

0.5 × 10^6^ HCT-116 cells were seeded in 35 mm petri dishes and treated with indicated concentration and time points of CPT and paclitaxel. After the treatment cells were processed for flowcytometry as described previously^51^. Propidium iodide fluorescence was acquired by BD FACS Calibur.

### siRNA knockdown experiments

siRNA targeting Vimentin was procured from Sigma-Aldrich (MISSION esiRNA, EHU151861) and transfection experiments were performed using lipofectamine 3000 (Life Technologies, Carlsberg, CA, USA) following manufacturer’s instructions.

### Luciferase reporter assay

To understand the effect of Vimentin signaling on NFκB expression, we employed NFκB promoter luciferase cassette (kind gift from Dr. Vivek M Rangnekar, University of Kentucky, Lexington, Kentucky) containing NFκB responsive element obtained from κ light chain enhancer placed upstream of SV-40 promoter in PGL2 (firefly luc) vector in two copies arranged in tandem^52^. 1 × 10^3^ HCT-116 cells transfected previously with NFκB-luc were seeded in a 96 well plate and transfection with siRNA for Vimentin was done. Subsequently, CPT treatment was given and the luciferase activity was measured with the help of a dual glow luciferase assay system (Promega, Madison, Wisconsin, United States) according to manufacturer’s instructions.

### Co-immunoprecipitation assay

Immunoprecipitation of both Vimentin and ATM kinase was performed following previously established method with minor modifications^51^. Briefly, 2.5 × 10^6^ HCT-116 cells were seeded in 90 mm petri dishes and CPT/vehicle treatment was given for 36 h. After the treatment, cells were harvested by gentle scrapping and washed with PBS; following centrifugation the pellets were suspended in RIPA buffer containing 1% Triton X-100, 5 mM EDTA, 0.1% SDS, 1% NP4, 1 mM PMSF, 100 µM Na_3_VO_4_, and 1% protease inhibitor cocktail (Roche, Basel, Switzerland). Subsequently, the cell lysates were centrifuged at 14,000 rpm/10 min to remove the debris. Pre-clearing was performed with 25 µl of Protein-G PLUS agarose beads (Santa-Cruz, Dallas, Texas, USA) and precleared lysates were further subjected to immunoprecipitation with 5 µg antibody conjugated to 50 µl of Protein-G PLUS agarose beads. The co-immunoprecipitated proteins were washed with RIPA buffer and further analyzed by western blotting.

### In vivo studies

All animal studies were done in accordance with the protocols prescribed by the Institute Animal Ethical Committee of CCMB (IAEC), Hyderabad. The *Apc* knockout model (*AhCre-ErT Apcfl/fl*) is an inducible colon cancer murine model having crypt progenitor phenotype in the intestine upon induction with β-naphthoflavone and tamoxifen, leading to progressive knockout and death within 7 to 10 days’ time. To analyze the effect of CPT-mediated DNA damage response to EMT in vivo, mice were divided into four different cohorts of three mice each including control group. Each cohort was further divided into two time points of 24 and 48 h. For inducing *Apc* knockout, intraperitoneal injections were given to 6 to 8-week-old mice with 80 mg/kg β-naphthoflavone and tamoxifen (dissolved in corn oil at 10 mg/ml) single dose per day till the end time point. Now respective cohorts were treated with Camptothecin, for that, oral gavaging of 10, 20 and 30 μg of CPT suspended in groundnut oil at 5 mg/ml was done, whereas control cohort received only groundnut oil. Thus, each mouse received 0.4, 0.8, 1.2 mg of CPT as per kg of body weight. Dosage of the drug was decided by the fact that the curing dose of CPT starts from 1 mg/kg. For combinatorial therapy, different cohorts of 3 mice each received 20 mg/kg DIM, 20 mg/kg DIM in combination with 0.4 mg/kg CPT and 0.4 mg/kg CPT suspended in groundnut oil along with vehicle (groundnut oil) orally. Following treatment, animals were sacrificed as per the ethical guidelines at specific end time points followed by cardiac perfusion and tissues collection. The tissues harvested were preserved accordingly for all downstream experiments. Intestines were specifically cleaned in running tap water, opened up and fixed in methacam for 48 h. The tissues were then made into a gut roll and preserved in 10% formalin for 48 h and after processing were sectioned at 4 μm thickness.

### Immunohistochemistry

Hematoxylin and Eosin (H & E) and Immunohistochemistry were performed as follows. Sections were deparaffinized in xylene, rinsed in ethanol, and rehydrated. Heat induced epitope retrieval was performed by boiling slides in 10 mM sodium citrate buffer pH 6.0 for 20 min at 100 °C and sections were cooled down at room temperature. Slides were then washed with PBS and blocked with 3% H_2_O_2_ in methanol for 20 min to quench endogenous peroxidase activity. Subsequent washes of water and PBS were carried out and slides were blocked with 4% BSA in 1X PBS for 1 h at room temperature. Slides were then incubated with primary antibodies Vimentin, p-Vimentin, NF-κB (p65), all at 1:100 dilutions at 4 °C overnight. The next day slides were washed three times with PBST (0.1% Triton-X) for 15 min each and incubated for two hours at room temperature with anti-rabbit HRP conjugated secondary IgG (Abcam ab97051). After incubation slides were again washed three times with PBST for 15 min each and liquid diaminobenzidine (DAB) (DAKO Envision) was used as a chromogenic agent for staining (1 min) and sections were counterstained with Mayer’s haematoxylin. Negative controls of IHCs were prepared for each set by replacing primary antibody with PBS.

### Statistical analysis

Data were represented as the mean ± s.d of at least three independent experiments and *p valu*e was calculated using GraphPad Prism software Version 5.0 (GraphPad Software, La Jolla, CA, USA). Student *t* test were performed to compare the respective differences. *P* ≤ 0.05 was consider as significant.

## Supplementary information

Supplementary Table 1

Supplementary Table 2

Supplementary Figures
