# Supplementary material for: Vimentin activation in early apoptotic cancer cells errands survival pathways during DNA damage inducer CPT treatment in colon carcinoma model
Source: Cell Death Dis. 2019 Jun 13;10(6):467. doi: 10.1038/s41419-019-1690-2 (PMC6565729; doi:10.1038/s41419-019-1690-2)
Supplement: Supplementary file 1 — Supplementary Table 1 [file 41419_2019_1690_MOESM1_ESM.docx]

**Table S1.** List of antibodies used

| **S No.** | **Name of Antibody** | **Manufacturer** | **Working Dilutions** |
| --- | --- | --- | --- |
| 1 | Vimentin | Santa Cruz Biotechnology | WB, 1:1000; ICC, 1:200;  IHC, 1:100 |
| 2 | p^Ser38^Vimentin | Santa Cruz Biotechnology | WB, 1:200; IHC, 1:100 |
| 3 | Snail-1 | Cell Signaling Technology | WB, 1:1000 |
| 4 | ATM | Santa Cruz Biotechnology | WB, 1:500 |
| 5 | β-catenin | Santa Cruz Biotechnology | WB, 1:1000 |
| 6 | E-cadherin | Santa Cruz Biotechnology | WB, 1:2000 |
| 7 | Actin | Sigma Aldrich | WB, 1:2000 |
| 8 | Caspase-3 | Cell Signalling Technology | WB, 1:1000 |
| 9 | PARP-1 | Santa Cruz Biotechnology | WB, 1:500 |
| 10 | MMP-2 | Santa Cruz Biotechnology | WB, 1:500 |
| 11 | NFκB | Santa Cruz Biotechnology | WB, 1:2000; IHC, 1:100 |
| 12 | pAKT | Santa Cruz Biotechnology | WB, 1:500 |
| 13 | AKT | Santa Cruz Biotechnology | WB, 1:2000 |
| 14 | Survivin | Santa Cruz Biotechnology | WB, 1:500 |
| 15 | cFLIP | Santa Cruz Biotechnology | WB, 1:1000, ICC, 1:200 |
| 16 | Bid | Cell Signalling Technology | WB, 1:500 |
| 17 | Bcl2 | Santa Cruz Biotechnology | WB, 1:1000 |
| 18 | Bax | Cell Signalling Technology | WB, 1:500 |
| 19 | Anti-rabbit IgG HRP conjugated | Sigma Aldrich | WB, 1:2000 |
| 20 | Anti-mouse IgG HRP conjugated | Sigma Aldrich | WB, 1:2000 |
| 21 | Anti-rabbit IgG Alexa Fluor 488^®^ conjugated | Thermo Fisher Scientific | WB, 1:1000 |

WB: Western blotting; ICC: Immunocytochemistry; IHC: Immunohistochemistry
