# Supplementary material for: Vimentin activation in early apoptotic cancer cells errands survival pathways during DNA damage inducer CPT treatment in colon carcinoma model
Source: Cell Death Dis. 2019 Jun 13;10(6):467. doi: 10.1038/s41419-019-1690-2 (PMC6565729; doi:10.1038/s41419-019-1690-2)
Supplement: Supplementary file 2 — Supplementary Table 2 [file 41419_2019_1690_MOESM2_ESM.docx]

**Table S2.** List of primers for Colony PCR

| **Primer code** | **Primer sequence** | ***T*m (◦C)** |
| --- | --- | --- |
| SecA5mVF | AAGAATTCATGGTGCACAAGGTGCTGGCC | 52 |
| SecA5mVR | AAGGATCCTTGTACAGCTCGTCCATGCC | 52 |

.
