# Supplementary material for: Vimentin activation in early apoptotic cancer cells errands survival pathways during DNA damage inducer CPT treatment in colon carcinoma model
Source: Cell Death Dis. 2019 Jun 13;10(6):467. doi: 10.1038/s41419-019-1690-2 (PMC6565729; doi:10.1038/s41419-019-1690-2)
Supplement: Supplementary file 3 — Supplementary Figures [file 41419_2019_1690_MOESM3_ESM.pdf]

Supplementary Information

Vimentin activation in early apoptotic cancer cells errands survival pathways during DNA damage inducer CPT treatment of colon carcinoma model

Souneek Chakraborty[1, 2], Aviral Kumar[3], Mir Mohd Faheem[2], Archana Katoch[1, 2], Anmol Kumar[3] Vijay Lakshmi Jamwal[4], Debasis Nayak[1, 2], Aparna Golani[3], Reyaz Ur Rasool[1, 2], Syed Mudabir Ahmad[1, 2], Jedy Jose[3], Rakesh Kumar[5], Sumit G Gandhi[4], Lekha Dinesh Kumar[3, *], Anindya Goswami[1, 2, *].

[1]Academy of Scientific & Innovative Research (AcSIR), CSIR-Indian Institute of Integrative Medicine, Jammu 180001, India

[2] Cancer Pharmacology Division, CSIR-Indian Institute of Integrative Medicine, Jammu 180001, India

[3] Cancer Biology, CSIR-Centre for Cellular & Molecular Biology, Hyderabad 500007, India

[4] Plant Biotechnology Division, CSIR-Indian Institute of Integrative Medicine, Jammu 180001, India

[5]School of Biotechnology, Shri Mata Vaishno Devi University, Katra 182320, India

Running title: Coexistence of EMT and apoptosis due to DNA damage responses

[*]**Corresponding Author:**

Anindya Goswami, PhD

CSIR- Indian Institute of Integrative Medicine,

Canal Road, Jammu 180001, India

Tel.: +91 0191 2569111; Fax: +91 01912569333

E-mail : agoswami@iiim.ac.in

Lekha Dinesh Kumar, PhD

CSIR- Centre For Cellular And Molecular Biology.

Uppal Road, Habsiguda, Hyderabad, Telangana 500007

Tel : +91040-27192933

E-mail : lekha@ccmb.res.in

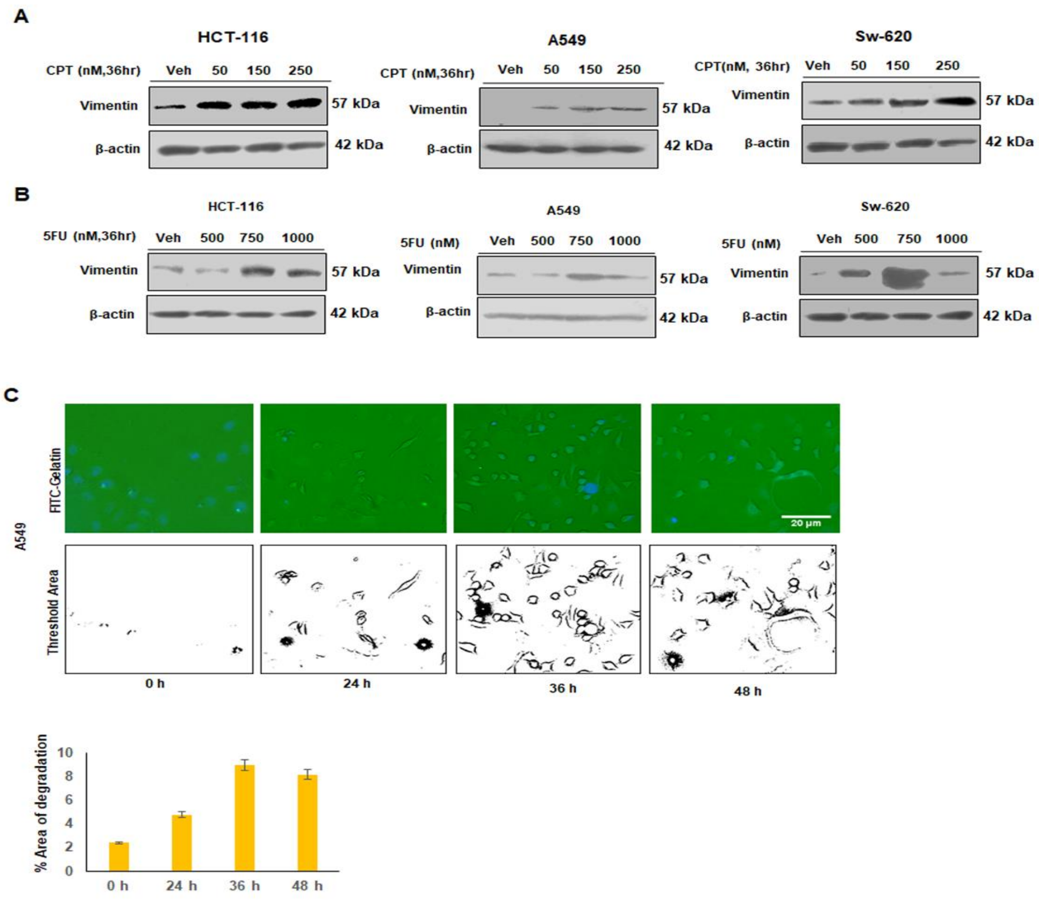

Supplementary Figure 1: Vimentin expression and invasion in response to various DNA damaging drugs. (A) HCT-116, A549 and Sw-620 cells were treated with indicated doses of CPT for 36 h and analyzed for Vimentin expression by western blotting. (B) Similar sort of experiment was carried with the indicated doses of 5-FU. β-actin was used as a loading control. (C) A549 cells, treated with CPT (250 nM) for 24, 36, 48 h along with vehicle and tested for their ability to degrade gelatin matrix through FITC-gelatin degradation assay. Blue stains indicate nuclear staining through DAPI mounting media. Images were taken at 20X magnification, scale bar: 100 µm. Bar graph showing the threshold area of degradation quantified through Image j analysis (*n* = 3, error bars ± s.d.)

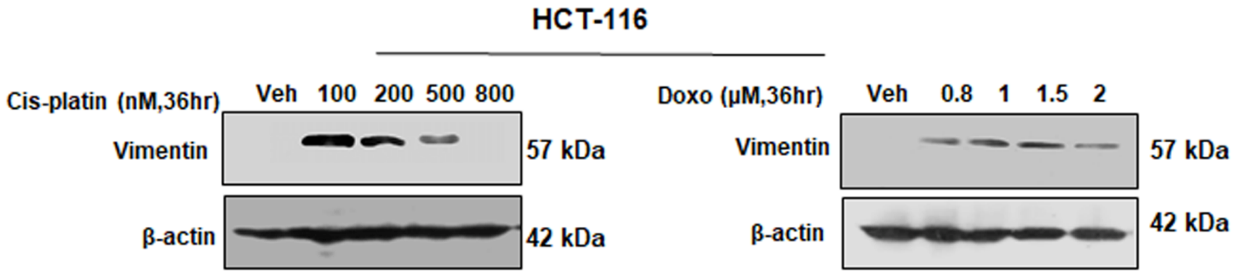

Supplementary Figure 2: Vimentin expression in Cis-platin and Doxorubicin treated HCT-116 cells. HCT-116 cells were treated with indicated doses of Cis-platin and doxorubicin for 36 h and analyzed for Vimentin expression by western blotting.

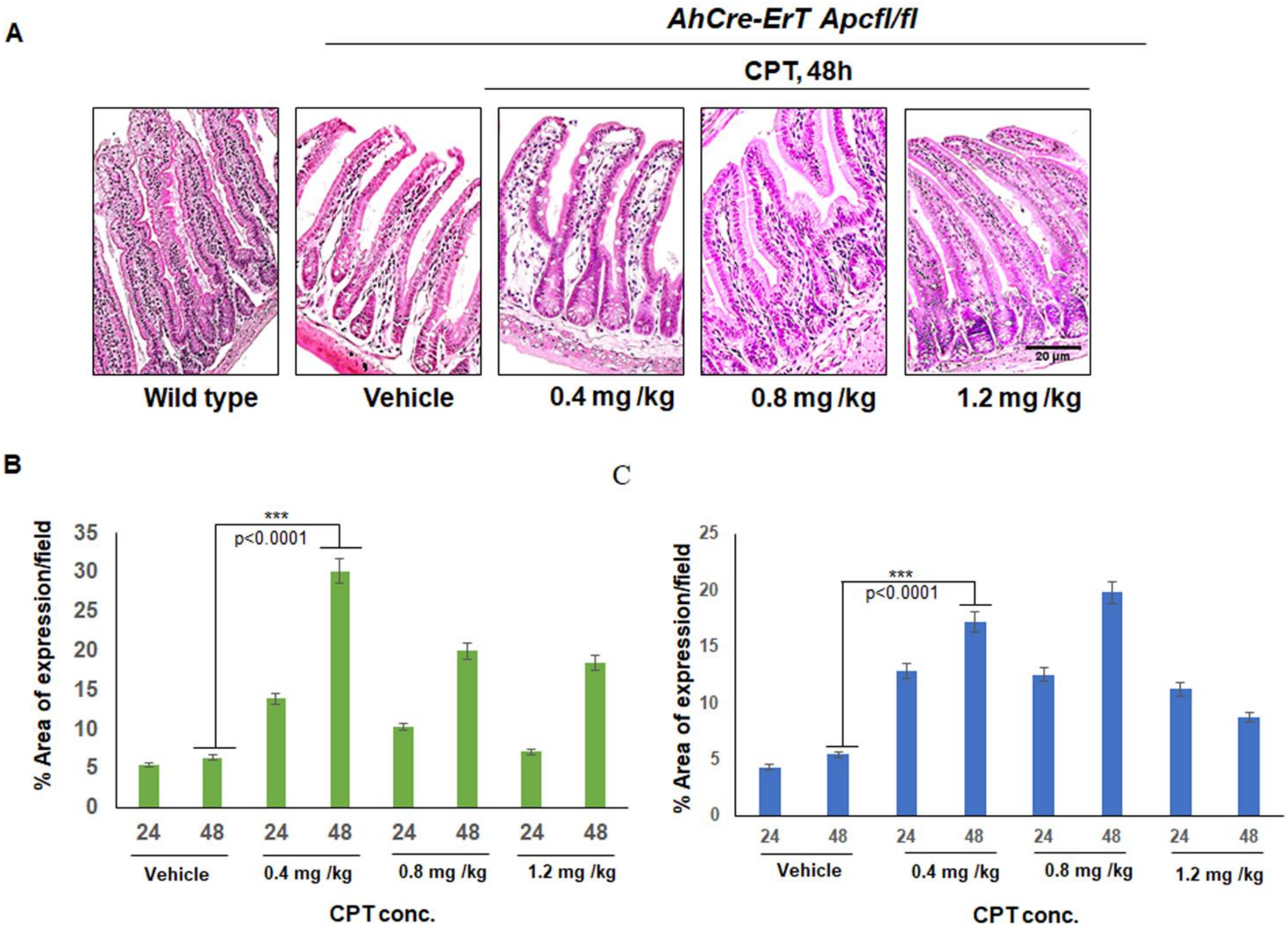

Supplementary Figure 3: CPT mediated activation of EMT and disruption of crypts in Apc floxed colorectal cancer model (A) Intestinal tissue obtained from induced *AhCre-ErT Apcfl/fl* treated with vehicle, 0.4 mg/kg, 0.8 mg/kg and 1.2 mg/kg along with wild type mice were stained with Hematoxylin & Eosin stain. (B-C) Bar graph represents the immunohistochemistry analysis of Vimentin and p^{ser38}Vimentin protein /field. Individual data points correspond to quantitative analysis of three independent images of labelled conditions by IHC toolbar attachment of Image j software; (*n* = 3, error bars ± s.d.); *** *p<0.0001*.

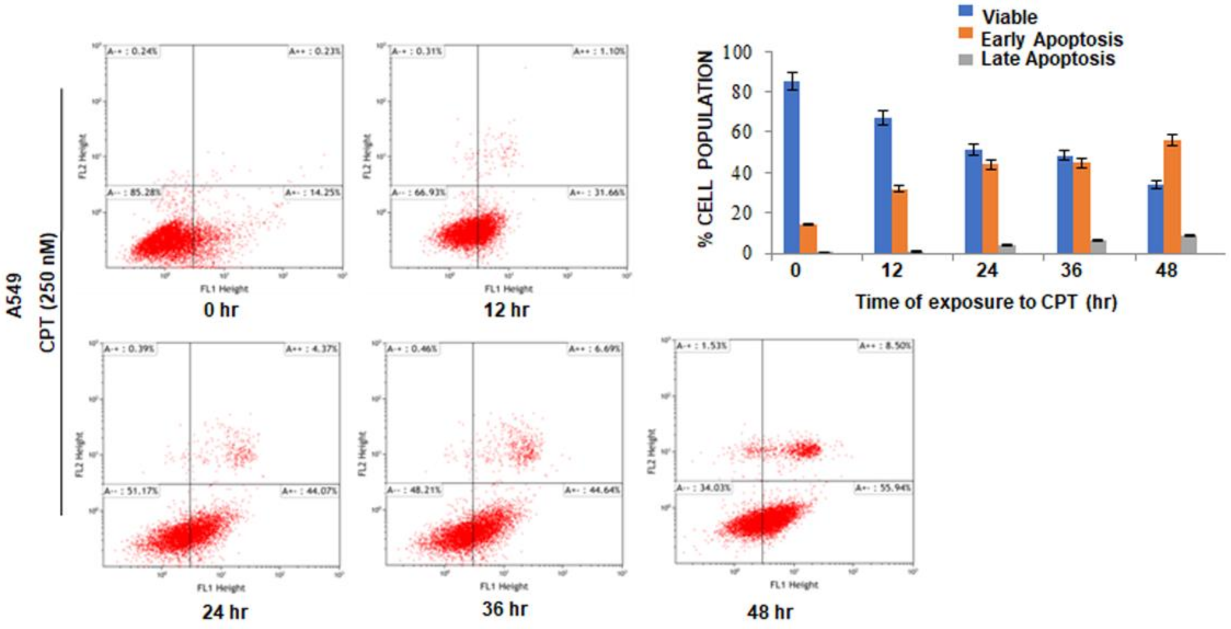

Supplementary Figure 4: Activation of Apoptosis in A549 Cells. A549 cells were treated with CPT for 0, 12, 24, 36, and 48 h, tagged with Annexin V-FITC, propidium iodide and analyzed through flow cytometry for onset of apoptosis. Bar graphs showing quantification of cells in various phases of apoptosis (*n* = 3, error bars ± s.d.).

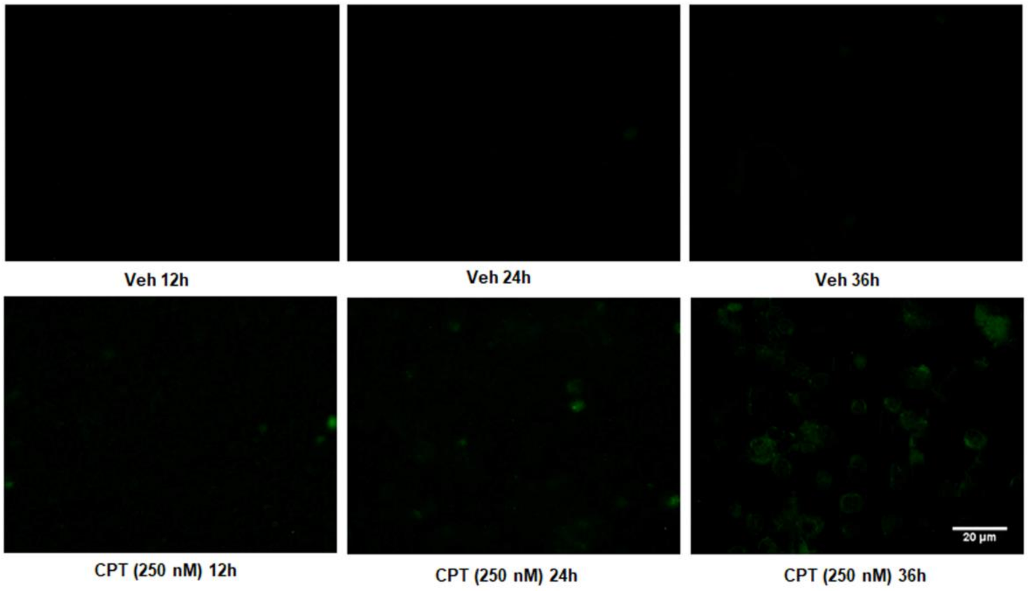

Supplementary Figure 5: **Evaluation of secreted *SecAnnexinV-mVenus* chimeric protein.** HCT cells seeded in 90mm petri dish, initially transfected with ***N3-secAnnexinV-mVenus*** plasmid and the conditional media was harvested after 48h. In another set of experiment HCT-116 cells were seeded in 6 well plate and CPT (250 nM)/vehicle treatment was given in the harvested conditional media for the indicated time periods. Images were obtained at 20X magnification.

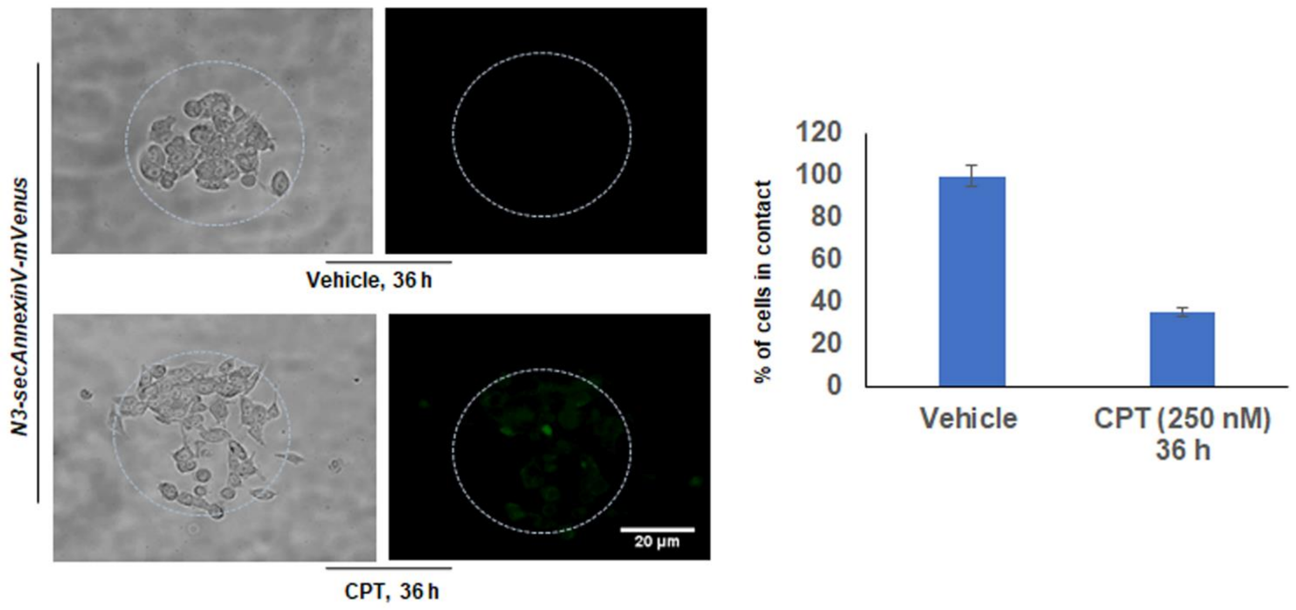

Supplementary Figure 6: Cell scattering analysis of *N3-secAnnexinV-mVenus* transfected cells. *N3-secAnnexinV-mVenus* transfected HCT-116 cells were employed for the cell scattering assay and treatment was given with CPT (250 nM) for 36 h. Scattering of cells along with expression of sec Annexin-mVenus was visualized under fluorescence microscope. Magnification 20X ; scale bar: 100µm. Bar graph represent percentage of cells that were in contact per field (n=3, error bars ± s.d).

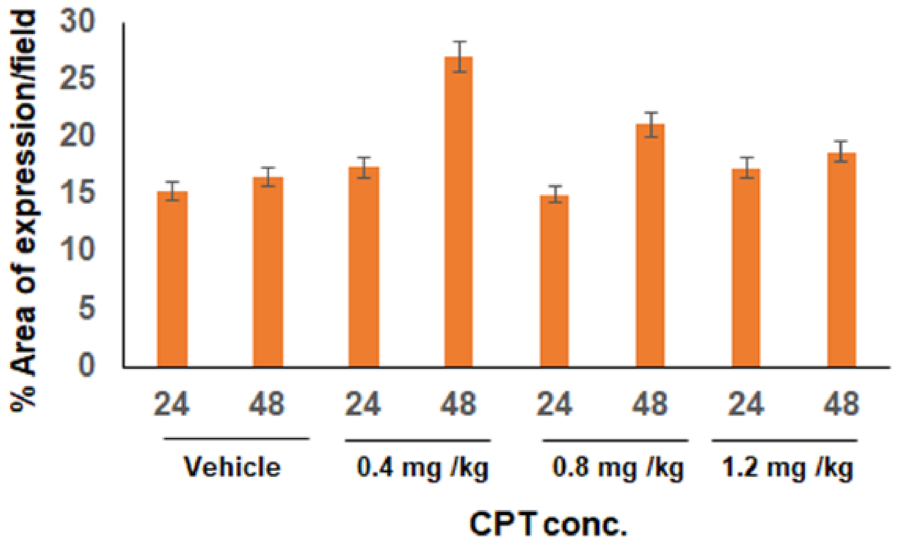

Supplementary Figure 7: Expression analysis of NFκB in *Apc* floxed model system. Bar graph represents the immunohistochemistry analysis of expression of NFκB protein /field. Individual data points correspond to quantitative analysis of three independent images of labelled conditions by IHC toolbar attachment of Image j software (n=3, error bars ± s.d).

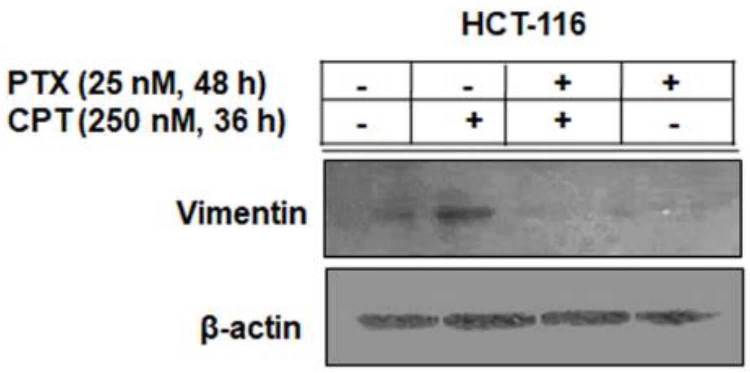

Supplementary Figure 8: Abrogation of Vimentin in G_2 arrested CPT treated cells. HCT-116 cells were treated with vehicle, CPT (250 nM), Paclitaxel (25 nM) + CPT (250 nM) and Paclitaxel (25 nM). Paclitaxel treatment was given 12 h before CPT treatment and the conditions were maintained upto 48h. The whole cell lysates were subjected to western blotting for determination of Vimentin expression.

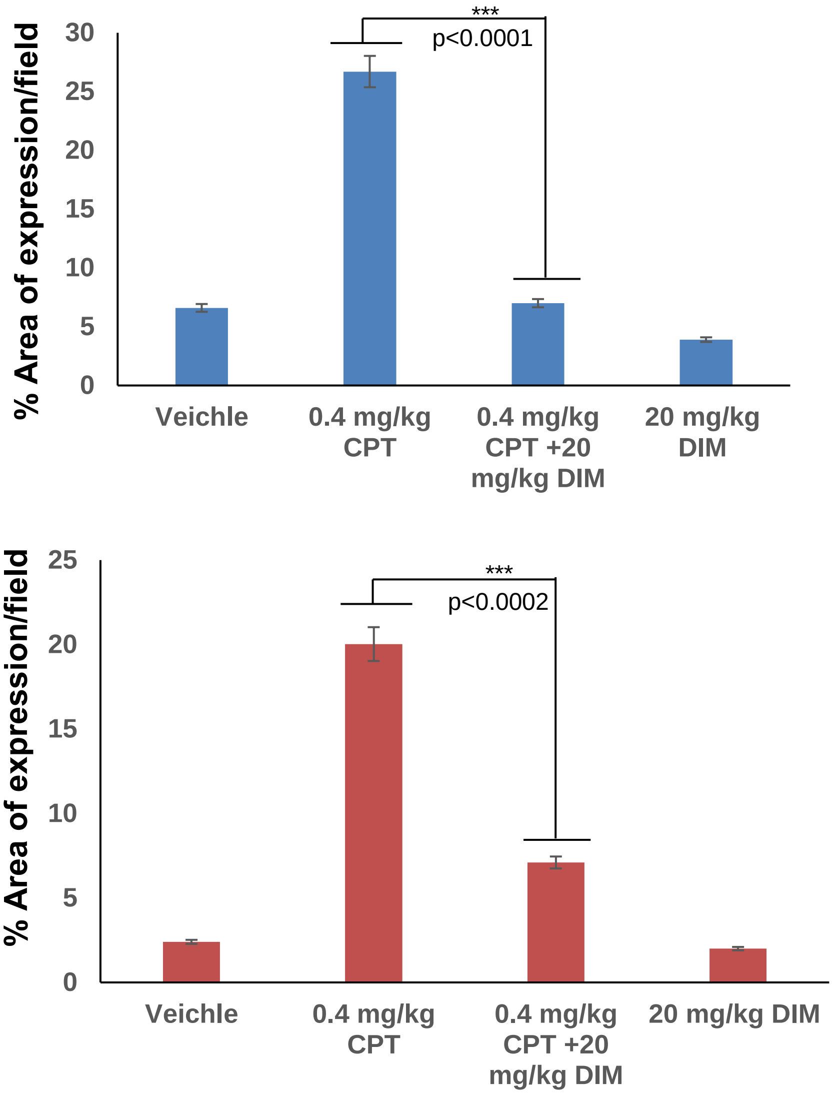

Supplementary Figure 9: Expression analysis of Vimentin and p^{ser38}Vimentin in *Apc* floxed model system. Bar graph represents the immunohistochemistry analysis of expression of both vimentin and p^{ser38}Vimentin protein /field. Individual data points correspond to quantitative analysis of three independent images of labelled conditions by IHC toolbar attachment of Image j software; (n = 3, error bars ± s.d.); *** *p<0.0001& p<0.0002.*
